# HIV Self-testing and Risk Behaviors Among Men Who Have Sex With Men in 23 US Cities, 2017

**DOI:** 10.1001/jamanetworkopen.2022.47540

**Published:** 2022-12-19

**Authors:** Cedric H. Bien-Gund, Pamela A. Shaw, Christine Agnew-Brune, Amy Baugher, Kathleen A. Brady, Robert Gross

**Affiliations:** 1 Division of Infectious Diseases, Department of Medicine, University of Pennsylvania Perelman School of Medicine, Philadelphia; 2 Kaiser Permanente Washington Health Research Institute, Seattle; 3 Division of HIV Prevention, Centers for Disease Control and Prevention, Atlanta, Georgia; 4 AIDS Activities Coordinating Office, Philadelphia Department of Public Health, Philadelphia, Pennsylvania; 5 Department of Biostatistics, Epidemiology, and Informatics, University of Pennsylvania Perelman School of Medicine, Philadelphia

## Abstract

**Importance:**

HIV self-testing (HIVST) is a promising strategy to expand the HIV care continuum, particularly among priority populations at high risk of HIV infection. However, little is known about HIVST uptake among men who have sex with men (MSM) outside of clinical trial settings.

**Objective:**

To evaluate HIVST use among urban MSM in the US who reported testing within the past 12 months.

**Design, Setting, and Participants:**

A cross-sectional study of adult MSM in the 2017 National HIV Behavioral Surveillance system, which used venue-based sampling methods to collect data related to HIV testing, receipt of prevention services, and risk factors for HIV, was conducted at 588 venues in 23 urban areas in the contiguous US and Puerto Rico. All participants were offered HIV testing. Adult cisgender MSM who reported HIV-negative or unknown HIV status and obtained HIV testing in the past 12 months were included. Data for this study were collected between June 4, 2017, and December 22, 2017, and analyzed between October 23, 2020, and August 20, 2021.

**Main Outcomes and Measures:**

Self-reported HIVST in the past year. Adjusted prevalence ratios (aPRs) using survey weights were calculated to assess factors associated with HIVST.

**Results:**

A total of 6563 MSM in 23 urban areas met inclusion criteria, of whom 506 (7.7%) individuals reported HIVST in the past year. The median age of self-testers was 29 (IQR, 25-35) years, 52.8% had completed college, and 37.9% reported non-Hispanic White race. One self-tester reported seroconverting in the prior 12 months, and an additional 10 self-testers were diagnosed with HIV during the survey. HIVST was associated with sexual orientation disclosure (aPR, 10.27; 95% CI, 3.45-30.60; *P* < .001), perceived discrimination against people with HIV (aPR, 1.53; 95% CI, 1.09-2.03; *P* = .01), younger age (aPR, 0.74; 95% CI, 0.66-0.84; *P* < .001), higher educational level (aPR, 1.20; 95% CI, 1.04-1.37; *P* = .01), and higher income levels (aPR, 1.18; 95% CI, 1.04-1.32; *P* = .009). No association was noted with condomless anal sex (aPR, 0.96; 95% CI, 0.88-1.06, *P* = .88), sexually transmitted infections (aPR, 0.96; 95% CI, 0.70-1.30; *P* = .77), or preexposure prophylaxis use (aPR, 0.99; 95% CI, 0.75-1.30; *P* = .92).

**Conclusions and Relevance:**

In this study, HIVST was relatively uncommon in this sample of urban MSM. HIVST may not be reaching those with lower socioeconomic status or who have not disclosed their sexual identity. The findings of this study suggest that efforts to increase HIVST should focus on engaging underserved and vulnerable subgroups of MSM.

## Key Points

**Question:**

Who is using HIV self-testing among men who have sex with men (MSM) in the US?

**Findings:**

In this cross-sectional study of 6563 MSM who reported HIV testing in the past year, 7.7% reported HIV self-testing within the past year. Self-testing was more common among younger MSM with higher socioeconomic status and those who reported sexual identity disclosure.

**Meaning:**

The findings of this study suggest that efforts to expand HIV self-testing among MSM may need to focus on reaching vulnerable subgroups, including those with lower socioeconomic status and who have not disclosed their sexual identity.

## Teaser

This cross-sectional study examines the use of HIV self-testing among men who have sex with men.

## Introduction

Testing for HIV is the critical first step in both HIV prevention and treatment efforts. Despite multiple interventions to increase HIV testing, novel strategies are needed to increase testing among men who have sex with men (MSM), who represent nearly two-thirds of all new HIV diagnoses in the US.^1^ One promising strategy to increase HIV test uptake is HIV self-testing (HIVST), whereby individuals self-collect and interpret a test result on their own. Despite interest in HIVST since the beginning of the HIV epidemic, a rapid HIVST method was not approved by the US Food and Drug Administration and was not available in the US until late 2012.^2,3,4^ Self-testing may be preferred by individuals who are reluctant to seek facility-based testing and may lead to increased awareness of HIV risk^5,6^ and partner testing.^7^ In study settings, self-testing has been associated with increased testing frequency and uptake among MSM.^8,9^ In a randomized trial evaluating internet-distributed HIV self-tests among MSM, self-testing identified more HIV infections than providing test information alone.^10^ In addition, studies have reported high feasibility and acceptability among MSM.^11,12,13,14^

However, little is known about HIVST uptake among MSM outside of clinical trial settings. Furthermore, it is unknown whether self-testing is associated with preventive behaviors and uptake of biomedical interventions, such as preexposure prophylaxis (PrEP). Understanding behaviors associated with self-testing will therefore be critical to informing implementation of HIV prevention efforts.

In this study, we examined HIV self-testing uptake among participants in a national survey of MSM in 23 urban areas across the contiguous US and Puerto Rico. We assessed prevalence of self-testing, characteristics associated with self-testing, and whether self-testing was associated with risk and preventive behaviors among MSM who reported HIV testing in the past 12 months and negative or unknown status. We hypothesized that HIV self-testing would be associated with increased testing and uptake of preventive services.

## Methods

### Study Design

We analyzed cross-sectional data from the Centers for Disease Control and Prevention (CDC) National HIV Behavioral Surveillance (NHBS) system conducted in 2017, which focused specifically on MSM across 23 urban areas, or metropolitan statistical areas, in the contiguous US and Puerto Rico.^15^ This surveillance project used venue-based sampling methods at sites frequented by MSM.^16^ Trained interviewers obtained informed consent and conducted face-to-face interviews, which collected data related to HIV testing behaviors, receipt of prevention services, and risk factors for HIV. In addition to completing a survey, all study participants were offered a rapid, blood-based HIV test. Each jurisdiction was allowed to select the specific type of test. If results of the first rapid test were positive or indeterminate, a second test was administered according to local public health testing procedures. These data were collected by state and local health departments and their partners with funding from the CDC. Data collection for the present study began on June 4, 2017, and was completed on December 22, 2017. This study was determined to be exempt from review by the CDC Human Research Protection Office and the University of Pennsylvania Institutional Review Board because data were collected for public health purposes and part of routine disease surveillance activity. All participants provided informed consent and were compensated for their services. This study followed the Strengthening the Reporting of Observational Studies in Epidemiology (STROBE) reporting guideline.

### Study Participants

Individuals were eligible for the NHBS system if they reported male sex at birth and current gender identity as being a man, being at least aged 18 years, having prior oral or anal sex with a male partner, and residing in a participating metropolitan statistical area. We further restricted our analyses to individuals who reported HIV testing in the past 12 months to limit confounding by indication. We excluded individuals who reported a diagnosis of HIV infection at least 12 months prior to the survey to exclude individuals without an indication for HIV testing in the past year.

### Study Measures

Our primary outcome was reported self-testing with a rapid HIV home test at least once in the past 12 months. Individuals who did not report self-testing with a rapid HIV home test at least once in the past 12 months were considered non–self-testers. We obtained self-reported HIV status at the time of the study, date of HIV diagnosis, and HIV testing data from the NHBS database.

Sociodemographic characteristics examined included age, income, educational level, and current insurance. We included race and ethnicity variables given the substantial racial and ethnic disparities in the HIV epidemic; participants self-reported Hispanic or Latino ethnicity (binary variable) and race (American Indian or Alaska Native, Asian, Black or African American, Pacific Islander, White, or multiracial). Sexual identity disclosure (“being out”) was a binary variable defined as telling anyone about their same-sex attraction or behavior. We obtained information on risk factors for HIV acquisition, including sexual behaviors, injection drug use, sexually transmitted infections, and information on preventive behaviors, including frequency of HIV testing, time of last HIV test, condom use, and PrEP knowledge and use. Individuals were considered to have seroconverted in the past year if they reported HIV-negative or unknown status 1 year prior to the survey and reported an HIV diagnosis date in the past 12 months prior to the survey. We defined new HIV diagnoses as those who reported HIV unknown or negative status at the time of the survey and subsequent positive rapid and confirmatory HIV tests during the NHBS survey. We defined male sex partners as having had oral or anal sex with another man. Condomless anal sex included both insertive and receptive anal sex. PrEP awareness and use were binary variables defined as having ever heard of PrEP prior to the survey, and PrEP use in the past 12 months, respectively. We also examined measures on discrimination and stigma associated with HIV and sexual identity, which may be barriers to accessing HIV testing services. Measures of perceived stigma and discrimination were based on agreement with the following statements: my community would (1) discriminate against people with HIV, (2) would not support the rights of people with HIV, (3) would not be friends with people with HIV, and (4) believe people with HIV got what they deserved. Participants answered on a 5-point Likert scale from 1 (strongly disagree) to 5 (strongly agree) and responses were coded as disagree/neutral (1-3) or agree (4-5). Measures of experienced discrimination due to sexual identity were binary variables and included (1) subjected to verbal discrimination, (2) received poor service, (3) treated unfairly at work or school, (4) received lower quality health care, or (5) was physically attacked or injured.

### Statistical Analysis

We obtained weighted percentages and 95% CIs for variables of interest and compared characteristics of self-testers and non–self-testers by using the Rao and Scott χ^2^ test. We calculated crude and adjusted prevalence ratios (aPRs) and 95% CIs using Poisson multivariable regression that accounted for the survey design. We used survey interview weights based on all venue-attending MSM in the NHBS sample. Data were weighted to account for unequal selection probabilities, multiplicity, and nonresponse bias, allowing us to infer our estimates to all venue-attending MSM in these cities. We used robust sandwich variance estimation to account for clustering of survey data by venue. In adjusted models, we selected covariates based on a priori interest and on prior literature review. We adjusted for age, race and ethnicity, and city and built separate models for each covariate and the primary outcome. We assessed for linear trends for ordinal covariates. We considered a 2-tailed α level of .05 to be statistically significant. All analyses were done in Stata, version 15.1 (StataCorp LLC).

## Results

A total of 10 760 individuals were surveyed in the NHBS system across 588 venues in 23 urban areas. Of these, 435 individuals (4.0%) had never tested for HIV, 1937 (18.0%) reported an HIV diagnosis more than 1 year ago, 1762 (16.4%) had their last HIV test done more than a year ago, and 63 (0.6%) did not have HIV testing data and were excluded from the study. A total of 6563 MSM reported HIV-negative or unknown status and testing for HIV in the past year and were included in the analysis (Figure).

**Figure.**
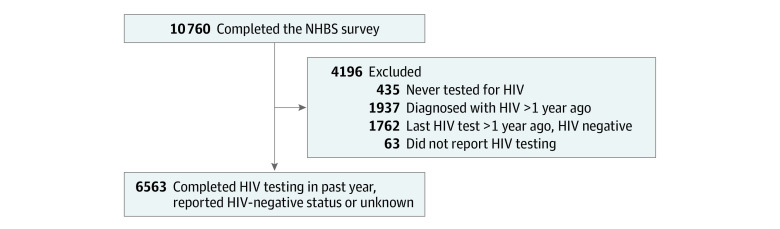
Flowchart of the Study Population From the 2017 National HIV Behavioral Surveillance (NHBS) System

Among the 6563 men included in the analysis, 506 individuals (7.7%) reported at least 1 HIV self-test in the past year. One self-tester reported being diagnosed with HIV in the past 12 months, compared with 114 non–self-testers who reported diagnosis of HIV in the past 12 months. An additional 10 individuals (2.0%) who self-tested tested positive for HIV during the NHBS survey, compared with 162 non–self-testers (Table 1). The remaining 495 self-testers who reported HIV-negative or unknown status tested negative at the time of the NHBS survey. Among those reporting self-testing, 313 individuals (61.9%) reported 1 self-test over the past year, 111 (21.9%) reported 2 self-tests, and 71 (14.0%) reported 3 to 5 self-tests in the past year. The median age of self-testers was 29 (IQR, 25-35) years, 52.8% had completed college, and 37.9% reported non-Hispanic White race. Among self-testers, the median number of reported male sex partners over the past 12 months was 5 (IQR, 3-12), and the median number of HIV tests in the past 2 years was 4 (IQR, 3-6).

**Table 1.** Baseline Characteristics of Men Who Have Sex With Men Reporting HIV-Negative/Unknown Status in 23 US Cities, 2017 (Total N = 6563)

Characteristic	HIV self-testing, No. (weighted %) [95% CI]^a^		*P* value^c^
	Reported (n = 506)^b^	None reported (n = 6057)^b^	
Age, y			
18-24	97 (19.7) [14.5-25.6]	1080 (19.1) [17.1-21.3]	<.001
25-34	274 (59.4) [52.7-65.7]	2665 (44.1) [41.2-46.2]	
35-44	84 (13.7) [9.8-18.8]	1152 (19.3) [17.8-21.0]	
45-54	43 (6.7) [4.1-10.8]	751 (11.6) [10.4-13.0]	
≥55	8 (1.0) [0.2-1.6]	409 (5.9) [4.8-7.1]	
Race and ethnicity			
Hispanic or Latino (any race)	137 (35.0) [28.9-41.6]	1585 (32.1) [29.9-34.5]	.52
Non-Hispanic Black	131 (23.6) [18.1-30.3]	1681 (23.6) [21.3-26.0]	
Non-Hispanic White	192 (30.9) [25.2-37.2]	2209 (35.4) [33.1-37.8]	
Other^d^	42 (10.5) [7.1-15.3]	546 (8.9) [7.9-10.0]	
Income, $			
0-12 499	47 (6.7) [4.1-10.8]	842 (11.8) [10.4-13.4]	.18
12 500-24 999	9 (2.7) [1.1-6.7]	219 (3.6) [2.9-4.5]	
25 000-49 999	194 (39.7) [32.9-46.8]	2278 (36.4) [34.3-38.6]	
50 000-74 999	96 (19.6) [15.0-25.3]	1128 (20.5) [18.9-22.3]	
≥75 000	157 (31.3) [25.1-38.2]	1541 (27.7) [25.7-29.7]	
Educational level			
High school or less	83 (13.2) [0.09-18.2]	1242 (18.7) [17.0-20.6]	.25
Some college	156 (33.6) [26.9-40.9]	1976 (32.1) [30.1-34.1]	
Completed college	164 (34.7) [28.3-41.6]	1891 (31.5) [29.6-33.6]	
More than college	103 (18.6) [14.0-24.4]	944 (17.7) [16.0-19.6]	
Has health insurance			
No	81 (13.3) [9.4-18.4]	998 (16.5) [15.0-18.2]	.20
Yes	423 (86.7) [81.6-90.6]	5047 (83.5) [81.8-85.0]	
Sexual identity disclosure to anyone			
No	7 (0.03) [0.01-0.8]	246 (4.1) [3.4-5.1]	<.001
Yes	499 (99.7) [99.1-99.9]	5809 (95.9) [95.0-96.7]	
No. of tests in past 2 y			
1-2	117 (25.4) [20.1-31.6]	1849 (30.0) [28.2-32.0]	.39
3-5	208 (40.7) [34.1-47.6]	2336 (40.1) [38.1-42.2]	
6-8	129 (22.5) [17.3-28.9]	1230 (21.2) [19.4-23.1]	
≥9	52 (11.4) [7.5-17.0]	509 (8.7) [7.5-9.9]	
Time of last HIV test			
7-12 mo ago	77 (16.5) [11.8-22.7]	1267 (20.7) [19.0-22.5]	.40
4-6 mo ago	112 (22.4) [17.4-28.5]	1314 (21.4) [19.8-23.1]	
≤3 mo	314 (61.0) [54.2-67.5]	3324 (57.9) [55.8-60.0]	
Discussed HIV prevention with outreach worker			
No	291 (61.3) [54.6-67.6]	4076 (69.3) [67.2-71.3]	.01
Yes	215 (38.7) [32.4-45.4]	1979 (30.7) [28.7-32.8]	
HIV diagnosis in past 12 mo			
No	505 (99.4) [95.4-99.9]	5943 (98.3) [97.7-98.7]	.31
Yes	1 (0.6) [0.09-4.7]	114 (1.8) [1.3-2.3]	
HIV diagnosis at time of survey			
No	496 (98.4) [96.2-99.3]^e^	5562 (97.6) [96.9-98.2]	.41
Yes	10 (1.6) [0.7-3.8]	162 (2.4) [1.8-3.1]	
Male sex partners, 12 mo			
0-1	69 (16.4) [11.9-22.3]	1151 (17.3) [15.6-19.1]	.44
2-4	137 (26.8) [21.3-33.2]	1727 (30.5) [28.5-32.6]	
5-7	83 (19.3) [14.0-26.0]	947 (15.4) [14.0-16.9]	
≥8	211 (37.5) [31.2-44.2]	2210 (36.8) [34.5-39.2]	
Condomless anal intercourse partners, in past 12 mo			
0	250 (51.8) [45.1-58.4]	3126 (50.8) [48.5-53.0]	.44
1	74 (14.1) [10.1-19.5]	877 (15.2) [13.8-16.8]	
2-4	98 (20.9) [15.8-27.1]	1023 (17.3) [15.8-18.9]	
5-7	25 (5.2) [3.0-9.1]	323 (5.4) [4.6-6.4]	
≥8	59 (8.0) [5.3-11.9]	708 (11.3) [10.0-12.8]	
Exchanged sex for drugs or money			
No	461 (94.3) [90.8-96.5]	5572 (93.0) [91.8-94.1]	.42
Yes	39 (5.7) [3.5-9.2]	461 (7.0) [6.0-8.2]	
Bacterial STI in past 12 mo			
No	397 (77.8) [71.9-82.7]	4784 (78.2) [76.3-80.0]	.88
Yes	107 (22.2) [17.3-28.1]	1269 (21.8) [20.0-23.7]	
PrEP awareness			
No	38 (5.7) [3.2-9.9]	612 (10.2) [8.8-11.7]	.04
Yes	410 (94.3) [90.1-96.8]	4634 (89.8) [88.2-91.2]	
PrEP use			
No	297 (68.3) [61.9-74.1]	3629 (68.2) [65.7-70.5]	.96
Yes	151 (31.7) [25.9-38.1]	1615 (31.8) [29.5-34.3]	

Abbreviations: PrEP, preexposure prophylaxis; STI, sexually transmitted infection.

^a^ Survey weights are based on all venue-attending men who have sex with men in the 2017 National Behavioral Health Surveillance system.

^b^ Numbers may not sum to total number and percentages may not total 100.0 due to missing data.

^c^ *P* values are from univariate χ^2^ test.

^d^ Includes individuals who reported as American Indian or Alaska Native, Asian, Pacific Islander, multiracial, and unknown race or ethnicity.

^e^ Includes the 1 person who was diagnosed in the past 12 months.

In our primary analysis, adjusted for age, race and ethnicity, and city, the prevalence of self-testing increased with educational level (aPR, 1.20; 95% CI, 1.04-1.37; *P* = .01) and income level (aPR, 1.18; 95% CI, 1.04-1.32; *P* = .009), and decreased with age (aPR, 0.74; 95% CI, 0.66-0.84; *P* < .001) (Table 2). We did not observe any association with race or ethnicity. Participants who had disclosed their sexual identity were 10.27 times more likely to report self-testing (95% CI, 3.45-30.60; *P* < .001). Regarding HIV testing, we did not observe any association of self-testing with increased rates of HIV testing, testing more recently, or new HIV diagnosis during the survey.

**Table 2.** Factors Associated With HIV Self-testing Among Men Who Have Sex With Men in the US, 2017 (N = 6563)

Characteristic	Crude PR (95% CI)^a^	*P* value	Adjusted PR (95% CI)^b^	*P* value
Age, y				
18-24	1 [Reference]	NA	1 [Reference]	NA
25-34	1.25 (0.91-1.74)	.17	1.20 (0.86-1.68)	.28^c^
35-44	0.70 (0.46-1.09)	.11	0.68 (43.9-1.06)	.09
45-54	0.58 (0.33-1.00)	.05	0.54 (0.30-0.96)	.04
≥55	0.10 (0.03-0.30)	<.001	0.11 (0.04-0.34)	<.001
Linear trend	0.75 (0.67-0.84)	<.001	0.74 (0.66-0.84)	<.001
Race and ethnicity				
Hispanic or Latino (any race)	1 [Reference]	NA	1 [Reference]	NA
Non-Hispanic Black	1.06 (0.74-1.51)	.76	1.04 (0.71-1.54)	.83
Non-Hispanic White	0.88 (0.63-1.23)	.45	0.91 (0.63-1.32)	.61
Other^d^	1.16 (0.72-1.87)	.54	1.13 (0.71-1.80)	.61
Income, $				
0-12 499	1 [Reference]	NA	1 [Reference]	NA
12 500-24 999	1.31 (0.50-3.40)	.58	1.12 (0.45-2.81)	.81^c^
25 000-49 999	1.81 (1.06-3.08)	.03	1.68 (1.00-2.83)	.05
50 000-74 999	1.63 (0.93-2.88)	.09	1.58 (0.90-2.77)	.13
≥75 000	1.90 (1.10-3.27)	.002	2.14 (1.23-3.70)	.003
Linear trend	1.11 (1.00-1.24)	.04	1.18 (1.04-1.32)	.009
Educational level				
High school or less	1 [Reference]	NA	1 [Reference]	NA
Some college	1.52 (0.99-2.33)	.06	1.46 (0.94-2.27)	.09^c^
Completed college	1.59 (1.05-2.39)	.03	1.62 (1.05-2.51)	.03
More than college	1.52 (0.97-2.39)	.07	1.85 (1.14-3.01)	.01
Linear trend	1.12 (0.99-1.26)	.08	1.20 (1.04-1.37)	.01
Has health insurance	1.26 (0.88-1.81)	.214	1.40 (0.97-2.02)	.07
Sexual identity disclosure	13.2 (4.50-38.9)	<.001	10.27 (3.45-30.60)	<.001
No. of tests in past 2 y				
1-2	1 [Reference]	NA	1 [Reference]	NA
3-5	1.16 (0.84-1.61)	.36	1.04 (0.75-1.45)	.80
6-8	1.23 (0.84-1.80)	.28	1.14 (0.77-1.70)	.51
≥9	1.49 (0.90-2.46)	.12	1.32 (0.80-2.18)	.29
Linear trend	1.13 (0.97-1.31)	.11	1.09 (0.93-1.27)	.28
Time of last HIV test				
7-12 mo ago	1 [Reference]	NA	1 [Reference]	NA
4-6 mo ago	1.28 (0.82-1.99)	.28	1.21 (0.78-1.89)	.39
≤3 mo	1.29 (0.87-1.91)	.2	1.22 (0.82-1.81)	.32
Test of trend	1.11 (0.93-1.33)	.24	1.09 (0.91-1.30)	.36
Discussed prevention strategies with an outreach worker	1.39 (1.08-1.80)	.01	1.27 (0.98-1.65)	.07
HIV diagnosis at time of survey	0.70 (0.29-1.67)	.42	0.71 (0.29-1.71)	.44
Male sex partners, 12 mo				
0-1	1 [Reference]	NA	1 [Reference]	NA
2-4	0.91 (0.60-1.37)	.65	0.84 (0.56-1.26)	.40
5-7	1.28 (0.82-2.01)	.27	1.15 (0.74-1.79)	.53
≥8	1.06 (0.72-1.58)	.76	0.99 (0.67-1.47)	.96
Linear trend	1.05 (0.93-1.18)	.42	1.03 (0.91-1.16)	.61
Condomless anal intercourse partners in the past 12 mo				
0	1 [Reference]	NA	1 [Reference]	NA
1	0.93 (0.63-1.38)	.72	0.46 (0.59-1.28)	.46
2-4	1.18 (0.84-1.67)	.35	1.17 (0.82-1.66)	.38
5-7	0.97 (0.54-1.73)	.91	0.94 (0.52-1.69)	.83
≥8	0.72 (0.46-1.11)	.14	0.72 (0.46-1.13)	.16
Linear trend	0.97 (0.89-1.05)	.42	0.96 (0.88-1.06)	.88
Exchanged sex for drugs or money	0.82 (0.51-1.34)	.44	0.89 (0.54-1.44)	.63
Bacterial STI in past 12 mo	1.03 (0.76-1.39)	.84	0.96 (0.70-1.30)	.77
PrEP use	0.99 (0.76-1.29)	.96	0.99 (0.75-1.30)	.92
PrEP awareness	1.80 (1.00-3.28)	.05	1.64 (0.88-3.05)	.12

Abbreviations: NA, not applicable; PR, prevalence rate ratio; PrEP, preexposure prophylaxis; STI, sexually transmitted infection.

^a^ Prevalence rate ratio and *P* values estimated from a univariate weighted Poisson model.

^b^ Prevalence rate ratio and *P* values estimated from separate weighted Poisson models adjusted for age, race and ethnicity, and city. National HIV Behavioral Surveillance survey interview weights were based on venue-attending men who have sex with men.

^c^ Global *P* value <.01.

^d^ Includes individuals who reported as American Indian or Alaska Native, Asian, Pacific Islander, multiracial, and unknown race or ethnicity.

Regarding associations with HIV risk behaviors, we did not observe an association with number of male sex partners, condomless anal sex, or other risk factors for HIV acquisition including exchanging sex for money or drugs or bacterial sexually transmitted infections (Table 2). We did not observe any association with PrEP awareness or PrEP use. Self-testing was higher among those who reported people in their community would not be friends with people with HIV (aPR, 1.53; 95% CI, 1.09-2.13; *P* = .01) but lower among those who reported verbal discrimination due to sexual identity (aPR, 0.80; 95% CI, 0.67-0.96; *P* = .02) (eTable in Supplement 1). We did not observe any significant differences with other measures of discrimination or stigma.

## Discussion

Widely available and flexible HIV testing options are critical to ending the HIV epidemic, and self-testing has emerged as a promising strategy to supplement standard facility-based testing efforts. We assessed HIV self-testing uptake in cross-sectional NHBS data of MSM with HIV-negative and unknown status at risk of HIV infection in 2017, which represents one of the largest samples of MSM at risk of HIV infection in the US. Uptake of self-testing was low and limited to roughly 1 in 13 MSM, and rates of self-testing were higher among younger, more educated, and wealthier MSM, and MSM who had disclosed their sexual identity.

Our observation that self-testing was more prevalent among younger, wealthier, more educated MSM raises questions regarding the implementation of HIVST to reach MSM experiencing poverty or with lower socioeconomic status. Although clinical trials have demonstrated benefits to offering HIVST, clinical trials present artificial environments outside of clinical settings and provided self-test kits free of charge. Prior research has suggested that price, marketing, and distribution strategies may have a substantial effect on uptake of HIVST.^17,18,19^ Our study suggests that additional interventions may be needed to increase HIVST access to individuals who are older or with lower incomes. For instance, research has suggested that HIVST distribution through social networks or peers may increase testing coverage.^10,20^ Implementation science approaches should focus on evaluating ongoing HIVST programs to optimize reach to priority populations.^21,22^

We also observed that MSM who had disclosed their sexual identity to anyone were 10 times more likely to self-test. Although most MSM sampled in the NHBS had disclosed their sexual identity to someone, this finding also suggests that additional implementation efforts are needed to extend HIVST to reach vulnerable MSM who have not yet disclosed their sexual identity. MSM who have not yet disclosed their sexual identity may face additional barriers to accessing needed sexual health and HIV services.^23,24^ However, we also found that self-testing was more common among MSM who perceived anti-HIV discrimination in their communities, who may prefer the privacy and anonymity associated with self-testing. Although clinical trials have demonstrated promise of HIVST to extend testing services to individuals who have no prior testing, our findings suggest that HIVST is still used mainly by MSM who have disclosed their sexual identity and are engaged in clinical care.

One concern with HIVST is that it may miss acute HIV infections when transmissibility is highest, prior to formation of antibodies that are detectable by current HIV self-tests.^4,25^ Current oral fluid-based HIVST has lower sensitivity compared with whole blood assays, and a false-negative test result may wrongly reassure individuals with HIV infection.^26^ In our study, 10 of the 506 individuals (2.0%) who reported self-testing and an HIV-negative or unknown status subsequently tested positive during the survey. Nonetheless, we did not observe a significant difference in HIV seropositivity between self-testers and non–self-based testers who reported a negative or unknown status. In addition, although self-testing was associated with discussing HIV prevention strategies with an outreach worker, we did not observe an association with PrEP awareness and use. These findings indicate the importance of frequent testing and supplementing HIV testing with other risk reduction strategies, such as PrEP.

### Strengths and Limitations

Our study has several strengths. The study draws on NHBS data from one of the largest surveillance surveys of MSM across multiple urban areas in the US, including Puerto Rico. Although literature on HIVST has been largely from randomized clinical trials, this survey presents data from a public health surveillance systems setting. The sample was racially diverse and represented a range of ages and socioeconomic status. Furthermore, although we collected self-reported HIV testing data, we were also able to verify self-reported HIV serostatus with HIV testing conducted through the NHBS system.

This research has limitations. First, our study was limited to MSM sampled at venues in urban areas and therefore may not be representative of all MSM in the US, such as MSM living in rural areas or those who use dating apps. This limitation is important to acknowledge because HIVST may be particularly useful for MSM using apps or those living in rural areas who may have less access to facility-based testing.^27^ However, this estimation represents one of the largest samples of MSM in the US. Second, although there was 1 self-tester who reported an HIV diagnosis in the past year, we were unable to confirm whether he was diagnosed during a self-test or a facility-based test. Third, because our study was cross-sectional, we could not evaluate longitudinal trends in HIV prevention services and behaviors. We were therefore unable to ascertain whether self-testers obtained PrEP after self-testing, used self-testing to monitor themselves on PrEP, or whether those who reported HIV diagnosis in the past 12 months identified their HIV infection through a self-test. Fourth, we were unable to assess how individuals obtained self-test kits and how much the test kits cost. Although we assessed whether MSM had contact with a prevention worker, we do not know whether MSM in the NHBS were also participants in other HIV prevention studies. Price and distribution strategy may influence whether individuals choose to use HIVST.

## Conclusions

Our findings suggest that there is an opportunity to expand implementation of HIVST in public health efforts to end the HIV epidemic. Furthermore, restrictions during the COVID-19 pandemic have demonstrated the promise of HIVST to provide more flexible and expansive testing options when access to facility-based testing is limited.^28,29^ Additional implementation research needs to focus on identifying ideal distribution strategies to underserved, vulnerable, and medically disconnected MSM, messaging to encourage test uptake, and linkage to care to further engage priority populations in the HIV prevention and care continuum.

## Notes

Supplement 1. **eTable.** Self-Testing and Measures of HIV Stigma and Discrimination Against Sexual Minorities Among MSM in 23 US Cities, 2017

Supplement 2. **Nonauthor Collaborators.** The NHBS Study Group

Supplement 3.
Data Sharing Statement
