# Supplementary material for: HIV Self-testing and Risk Behaviors Among Men Who Have Sex With Men in 23 US Cities, 2017
Source: JAMA Netw Open. 2022 Dec 19;5(12):e2247540. doi: 10.1001/jamanetworkopen.2022.47540 (PMC9856873; doi:10.1001/jamanetworkopen.2022.47540)
Supplement: Supplement 1. — eTable. Self-Testing and Measures of HIV Stigma and Discrimination Against Sexual Minorities Among MSM in 23 US Cities, 2017 [file jamanetwopen-e2247540-s001.pdf]

Supplementary Online Content

Bien-Gund CH, Shaw PA, Agnew-Brune C, Baugher A, Brady KA, Gross R; NHBS Study Group. HIV self-testing and risk behaviors among men who have sex with men in 23 US cities, 2017. *JAMA Netw Open*. 2022;5(12):e2247540. doi:10.1001/jamanetworkopen.2022.47540

eTable. Self-Testing and Measures of HIV Stigma and Discrimination Against Sexual Minorities Among MSM in 23 US Cities, 2017

This supplementary material has been provided by the authors to give readers additional information about their work.

© 2022 Bien-Gund CH et al. *JAMA Network Open.*

eTable: Self-Testing and measures of HIV stigma and discrimination against sexual minorities among MSM in 23 US cities, 2017

Factor	Adjusted PR (95% CI)[3]	P-value
Most people in my community would: Discriminate against PWH[1]		
Disagree/Neutral	Ref	
Agree	0.81 (0.61-1.07)	0.15
Would not the support of rights of PWH		
Disagree/Neutral	Ref	0.62
Agree	0.90 (0.55-1.4)	
Would not be friends with PWH		
Disagree/Neutral	Ref	0.01
Agree	1.53 (1.09-2.13)	
Belief PWH got what they deserved		
Disagree/Neutral	Ref	0.80
Agree	0.96 (0.70-1.32)	
Experienced verbal discrimination[2]		
No	Ref	0.02
Yes	0.80 (0.67-0.96)	
Discrimination – Poor Service		
No	Ref	0.22
Yes	1.14 (0.92-1.41)	
Discrimination – Work/school		
No	Ref	0.23
Yes	1.14 (0.92-1.42)	
Discrimination – Healthcare		
No	Ref	0.45
Yes	1.20 (0.74-1.95)	
Discrimination – Physical Assault		
No	Ref	0.43
Yes	0.87 (0.63-1.22)	

[1] HIV stigma measures were on a five point Likert scale from 1 (Strongly Disagree) to 5 (Strongly Agree) and coded as Disagree/Neutral (1-3) or Agree (4-5).
[2] Experiences of discrimination due to sexual identity measures were binary, Yes or No.
[3] Prevalence rate ratio (PR) and P-values estimated from separate weighted Poisson models adjusted for age, race/ethnicity, and city. NHBS survey interview weights were based on venue attending MSM.

© 2022 Bien-Gund CH et al. *JAMA Network Open.*
