# Supplementary material for: HIV Self-testing and Risk Behaviors Among Men Who Have Sex With Men in 23 US Cities, 2017
Source: JAMA Netw Open. 2022 Dec 19;5(12):e2247540. doi: 10.1001/jamanetworkopen.2022.47540 (PMC9856873; doi:10.1001/jamanetworkopen.2022.47540)
Supplement: Supplement 2. — Nonauthor Collaborators. The NHBS Study Group [file jamanetwopen-e2247540-s002.pdf]

Supplemental Online Content: Nonauthor Collaborators
*First name, last name, and suffix (if applicable) are required and will appear in PubMed.

*Group Name(s): NHBS Study Group							
***First Name and Middle Initial(s)**	***Last Name**	***Suffix (eg, Jr, III)**	Academic Degrees	Institution	Location (city, state/province, country)	Role or Contribution, eg, chair, principal investigator	Group (if more than 1 Group listed in the byline) and/or Subgroup (eg, Steering Committee)
Pascale	Wortley						
Jeff	Todd						
David	Melton						
Colin	Flynn						
Danielle	German						
Monina	Klevens						
Rose	Doherty						
Conall	O'Cleirigh						
Stephanie M.	Schuette						
David	Kern						
Antonio D.	Jimenez						
Jonathon	Poe						
Margaret	Vaaler						
Jie	Deng						
Alia	Al-Tayyib						
Melanie	Mattson						
Vivian	Griffin						
Emily	Higgins						
Mary-Grace	Brandt						
Salma	Khuwaja						
Zaida	Lopez						
Paige	Padgett						
Ekow Kwa	Sey						
Yingbo	Ma						
Shanell L.	McGoy						
Meredith	Brantley						
Randi	Rosack						
Emma	Spencer						
Willie	Nixon						

Supplemental Online Content: Nonauthor Collaborators
*First name, last name, and suffix (if applicable) are required and will appear in PubMed.

***First Name and Middle Initial(s)**	***Last Name**	***Suffix (eg, Jr, III)**	Academic Degrees	Institution	Location (city, state/province, country)	Role or Contribution, eg, chair, principal investigator	Group (if more than 1 Group listed in the byline) and/or Subgroup (eg, Steering Committee)
David	Forrest						
Bridget	Anderson						
Ashley	Tate						
Meaghan	Abrego						
William T.	Robinson						
Narquis	Barak						
Jeremy M.	Beckford						
Sarah	Braunstein						
Alexis	Rivera						
Sidney	Carrillo						
Barbara	Bolden						
Afework	Wogayehu						
Henry	Godette						
Kathleen A.	Brady						
Chrysanthus	Nnumolu						
Jennifer	Shinefeld						
Sean	Schafer						
E. Roberto	Orellana						
Amisha	Bhattari						
Anna	Flynn						
Rosalinda	Cano						
H. Fisher	Raymond						
Theresa	Ick						
Sandra Miranda	De León						
Yadira	Rolón-Colón						
Tom	Jaenicke						
Sara	Glick						
Celestine	Buyu						
Toyah	Reid						
Karen	Diepstra						
Jenevieve	Opoku						

Supplemental Online Content: Nonauthor Collaborators
*First name, last name, and suffix (if applicable) are required and will appear in PubMed.

***First Name and Middle Initial(s)**	***Last Name**	***Suffix (eg, Jr, III)**	Academic Degrees	Institution	Location (city, state/province, country)	Role or Contribution, eg, chair, principal investigator	Group (if more than 1 Group listed in the byline) and/or Subgroup (eg, Steering Committee)
Irene	Kuo						
Monica	Adams						
Chrstine	Agnew Brune						
Qian	Anderson						
Alexandra	Balaji						
Dita	Broz						
Janet	Burnett						
Johanna	Chapin-Bardales						
Melissa	Cribbin						
YenTyng	Chen						
Paul	Denning						
Katherine	Doyle						
Teresa	Finlayson						
Senad	Handanagic						
Brooke	Hoots						
Wade	Ivy						
Kathryn	Lee						
Rashunda	Lewis						
Lina	Nerlander						
Evelyn	Olansky						
Gabriela	Paz-Bailey						
Taylor	Robbins						
Catlainn	Sionean						
Amanda	Smith						
Anna	Templinskaya						
Lindsay	Trujillo						
Cyprian	Wejnert						
Akilah	Wise						
Mingjing	Xia						
