# Supplementary material for: HIV Self-testing and Risk Behaviors Among Men Who Have Sex With Men in 23 US Cities, 2017
Source: JAMA Netw Open. 2022 Dec 19;5(12):e2247540. doi: 10.1001/jamanetworkopen.2022.47540 (PMC9856873; doi:10.1001/jamanetworkopen.2022.47540)
Supplement: Supplement 3. — Data Sharing Statement [file jamanetwopen-e2247540-s003.pdf]

Data Sharing Statement

Bien-Gund. HIV Self-testing and Risk Behaviors Among Men Who Have Sex With Men in 23 US Cities, 2017. *JAMA Netw Open*. Published December 19, 2022. doi:10.1001/jamanetworkopen.2022.47540

Data
Data available: Yes
Data types: Data dictionary
How to access data: https://www.cdc.gov/hiv/statistics/systems/nhbs/methods-questionnaires.html
When available: With publication

Supporting Documents
Document types: None

Additional Information
Who can access the data: Researchers whose proposed use of the data has been approved and sponsored by any of the NHBS sites and subsequently approved by the national NHBS team.
Types of analyses: Researchers can propose analyses to NHBS, which if approved, a restricted dataset can be made available.
Mechanisms of data availability: Data can be made without investigator support, by contacting NHBS study site directors directly and obtaining local site support.
